# Effect of Sodium Hypochlorite Concentration in Continuous Chelation on Dislodgement Resistance of an Epoxy Resin and Hydraulic Calcium Silicate Sealer

**DOI:** 10.3390/polym13203482

**Published:** 2021-10-11

**Authors:** Nidambur Vasudev Ballal, Amal Roy, Matthias Zehnder

**Affiliations:** 1 Department of Conservative Dentistry and Endodontics, Manipal College of Dental Sciences, Manipal, Manipal Academy of Higher Education, Manipal 576104, India; drballal@yahoo.com (N.V.B.); amalroy999@yahoo.co.in (A.R.); 2 Clinic of Conservative and Preventive Dentistry, University of Zurich, 8032 Zurich, Switzerland

**Keywords:** root canal irrigants, root canal obturation, root canal filling materials

## Abstract

The conditioning of the root canal wall during chemo-mechanical root canal treatment differentially affects the adhesion of root canal sealers. This investigation evaluated the impact of sodium hypochlorite (NaOCl) concentration as used in a root canal irrigation concept called continuous chelation, with 1-hydroxyethylidene-1,1-diphosphonic acid (HEDP) contained in the NaOCl solution that is applied. Fourier-transform infrared spectra of the dentinal wall were gathered. The consequential effects on push-out bond strength of an epoxy resin (AH Plus) versus a hydraulic CaSi sealer (BioRoot RCS) were assessed. Single-rooted extracted human teeth were used and irrigated with pure NaOCl at a concentration of 0% (physiological saline), 2.5%, or 5.25%. Dual Rinse HEDP (9%) was added to the solutions, or not added for further control. Pure NaOCl solutions caused a decrease in the amide III: phosphate ratios, which was counter-acted by the addition of HEDP. It was observed that the adhesion of the epoxy resin sealer under investigation was negatively affected by this NaOCl deproteinization of the canal wall in a dose-dependent manner, while the opposite was observed with the CaSi sealer. HEDP when used in conjunction with NaOCl was beneficial for the adhesion of both sealers.

## 1. Introduction

Instrumentation, disinfection and three dimensional obturation of the root canal system are the crucial factors for successful endodontic treatment. During mechanical instrumentation of the root canal, a smear layer is formed on the radicular dentin and clogs the orifices of the dentinal tubules [1]. This smear layer can contain bacteria and prevent antimicrobial agents and sealants from gaining access to underlying contaminated dentinal tubules [2,3]. An alternating application of ethylenediaminetetraacetic acid and sodium hypochlorite (NaOCl) is frequently recommended for the effective removal of smear layer from the root canal system [4]. Though EDTA is widely used in daily clinical practice, it has several disadvantages such as cytotoxicity, reduced bonding of resin sealers, reduced smear layer removal efficacy in apical third, loss of available chlorine when mixed with NaOCl, and precipitate formation with chlorhexidine [5,6,7,8,9]. HEDP also known as etidronic acid, (1-hydroxyethylidene-1,1-diphosphonic acid) is a “soft” chelator that can be used in direct combination with NaOCl to form an all-in-one disinfecting, deproteinizing and chelating irrigant, which renders alternating rinses using chemically non-compatible solutions redundant. HEDP is less aggressive on dentin than EDTA and, hence, has a weak chelating capacity [4,10]. However, rather than removing a smear layer that has already formed, the continuous application of a combined solution of HEDP and NaOCl inhibits smear layer formation during instrumentation, hence the term “continuous chelation” [11,12]. It has also shown to reduce the accumulation of hard tissue debris in the isthmus area [13]. The combination of HEDP and NaOCl is advantageous in that this solution maintains its tissue dissolution capacity [11] and also does not affect NaOCl cytotoxicity [14].

Dual Rinse (9% HEDP) (Medcem, GmbH, Weinfelden, Switzerland) is a medical device (product approve for use in the root canal) based on this chemistry. It comes in a capsule containing 0.9 g of etidronate powder, which should be mixed immediately with fresh 10 mL of NaOCl solution of choice directly before treatment [15]. Based on the findings delineated above, the manufacturers claim that Dual Rinse HEDP conditions the root canal wall for subsequent obturation. However, as this etidronate powder is a mere add-on for the NaOCl solution/concentration of a clinician’s choice, the role of the NaOCl in this combination becomes an issue. NaOCl has a dose-dependent effect on the root canal wall in that it has a deproteinizing effect [16].

A root canal sealer plays an important role in the formation of a bond between root filling material and root canal dentin and also obtains a bacteria-tight seal of the root canal system [17]. As the currently used root filling materials do not effectively seal and resist ingress of microorganisms [18], augmentation of the root canal seal is also a desirable clinical goal because it may improve the outcome of the root canal treatment [19]. Epoxy resin-based sealers such as AH Plus (Dentsply DeTrey, Konstanz, Germany) have been widely used because of their acceptable physical properties, reduced solubility, apical sealability, adhesiveness to root dentin, and adequate biological performance [20,21]. More recently, sealers based on tricalcium silicate or other calcium silicate formulations were introduced due to their superior biocompatibility and bioactivity, and their ability to set in a wet environment [22]. Because the latter property, these materials, which are sometimes erroneously called bioceramic sealers, have been named hydraulic CaSi sealers. BioRoot RCS (Septodont, Saint Maur des Fosses, France) is one of the latest hydraulic CaSi sealers available to the dentist, and to the best of our knowledge the only one that incorporates a liquid phase for controlled hydration.

Dislodgement resistance also called push-out bond strength (POBS) is regarded as a pertinent prognostic factor to evaluate the association of a root canal sealer to the canal wall [23]. It is known that the adhesion of sealers is influenced by the chemical treatment performed to root canal dentin [24]. Epoxy resin-based sealers such as AH Plus bond to root canal dentin by formation of a covalent bond between its open epoxide ring and amino groups from exposed collagen in the root canal dentin [25]. In contrast, BioRoot RCS as a tri-calcium silicate-based sealer forms a so-called mineral infiltration zone with the dentin surface, i.e., a chemical bond to CaP, possibly combined with micromechanical interlocking through the formation of cement tags in the dentinal tubules [26]. This sealer is able to nucleate carbonated apatite related to its ability to release calcium ions and maintain an alkaline environment [27,28,29]. Difference in dentin conditioning may affect the performance of root canal sealers differently in clinical scenarios. Hence, to improve the capacity of enabling lesser microleakage along the sealer-dentin interface, irrigation protocols must be monitored [30]. It has been demonstrated that the continuous chelation irrigation with HEDP protocol optimizes the bond strength of an epoxy resin sealer to dentin [31]. However, hitherto, no studies have evaluated the effect of different concentrations of NaOCl with Dual Rinse HEDP on the POBS of BioRoot RCS and AH Plus sealers to root canal dentin. Hence, the objectives of this study were to evaluate the effect of different concentrations of NaOCl alone and in combination with Dual Rinse HEDP on the organic content in the root canal wall as assessed by Fourier-transform infrared spectroscopy (FTIR) [32], and the thus-resulting POBS of BioRoot RCS and AH Plus sealers to root canal dentin. The null hypotheses tested were (i) NaOCl concentration in the irrigating solutions do not affect amide III: phosphate ratios; and (ii), that there was no difference in the push out resistance between the sealers to root canal dentin.

## 2. Materials and Methods

### 2.1. Ethics and Teeth

The study protocol was approved by the Institutional Review board (IEC 768/2018) for the use of human teeth extracted according to individual treatment plans, which had nothing to do with this investigation. Soft tissue fragments and calcified debris on the specimens were removed using ultrasonic scalers. The specimens were stored in 0.2% sodium azide solution (Sigma-Aldrich, Steinheim, Germany) at 4 °C until use.

### 2.2. Treatment Groups and Sample Size Estimation

To study the influence of NaOCl concentration in continuous chelation, one capsule of Dual Rinse HEDP (Medcem) was freshly dissolved in 10 mL of physiological saline (Otsuka Pharmaceutical Inida Private Limited, Ahmedabad, India), 2.5% NaOCl, or 5.25% NaOCl before each experiment. These solutions without the HEDP were used as controls, resulting in six treatment groups.

The number of samples included in this study was based on the amide III: phosphate ratios in dentin after 30 min of exposure [33] and the POBS of epoxy resin-based sealer to root canal dentin treated with different irrigants [34], respectively. An effect size of >2 was assumed in both experiments. With an alpha error probability of 0.05, and 80% power (G*Power 3.1, Heinrich Heine Universität Düsseldorf, Düsseldorf, Germany) this resulted in 12 samples per group (*n* = 12). However, because of the qualitative rather than quantitative nature of this data, the sample size in the FTIR experiment was reduced to *n* = 5.

### 2.3. Fourier-Transform Infrared Spectroscopy (FTIR)

Thirty root canal dentin slices with dimensions of 1 cm × 1 cm were obtained from the root canal of extracted human single rooted teeth. The dentin slices were obtained from the middle third of each root. The root canal dentin surface of each specimen was polished using silicon carbide abrasive paper of 120 grit (Katyani Abrasives & Paints Pvt. Ltd., Ghaziabad, UP, India) and alumina suspensions. After attaining a flat and smooth surface that favours the absorbance of infrared radiation, the specimens were immersed in distilled water in ultrasonic bath for 1 min to remove the residues from polishing. The specimens were dried with absorbent paper to avoid excessive dehydration to reproduce tissue characteristics found in the clinical environment. The compositional analysis of all specimens was performed using the FTIR spectrophotometer (JASCO, Deutschland GmbH, Pfungstadt, Germany) with a diamond attenuated total reflection (ATR) set-up. The specimens were then positioned with the polished surface in contact with the diamond crystal of the ATR set-up and the initial FTIR spectra of each sample was recorded. Subsequently, the specimens were divided into six groups (*n* = 5) based on irrigation solutions used (pure 2.5% NaOCl, pure 5.25% NaOCl, 2.5% NaOCl + Dual Rinse HEDP, 5.25% NaOCl + Dual Rinse HEDP, saline + Dual Rinse HEDP, physiological saline). Because the etidronate powder (Dual Rinse HEDP) was directly dissolved in the NaOCl or saline solution as recommended by the manufacturer, there was only one solution per group. The specimens were placed inside microtubes containing 10 mL of either of these solutions for 30 min [35] in an orbital shaker (Umiya Biotech, Delhi, India). Subsequently, to remove the irrigants, the specimens were transferred to microtubes containing 10 mL of distilled water for 10 min. Then, the specimens were dried with absorbent paper, and a new infrared spectrum was recorded. The infrared bands considered were amide III, phosphate and carbonate. The areas under the considered bands were determined after the baseline tracing and the values obtained were used to calculate the amide III: phosphate and carbonate: phosphate ratios and the relative alterations in the contents of inorganic and organic components of dentin. Spectra was obtained between 400 and 4000 cm^−1^ at 4 cm^−1^ resolutions by using 64 scans per measurement.

### 2.4. Push-Out Bond Strength

A total of 144 single-rooted human teeth was selected for this experiment assessing six irrigants, 12 specimens per irrigant, and two sealers (one tooth per observation). Radiographs of the specimens were taken from buccal and mesial aspect to confirm a straight, single canal with mature apices and without any calcifications. The teeth were decoronated using a diamond disc (Horico, Berlin, Germany) and working length was established by inserting a size 10 K file (Mani Inc., Utsunomiya, Tochigi Ken, Japan) into each root canal until it was just visible at the apical foramen (observed using magnifying loupes, EyeMag Smart, Carl Zeiss, Oberkochen, Germany) then subtracting 1 mm from the recorded length. Apices of all the teeth were sealed with sticky wax to prevent the flow of irrigants through the apex and to allow an effective reverse flow of the irrigant, thereby simulating a closed end system. The specimens were then randomly divided into two times six groups (one group per irrigant and sealer, *n* = 12) based on the irrigation regimen (see above). Root canals were irrigated with 5 mL of irrigant after each instrument change during instrumentation, and the then again with 5 mL of that irrigant as a final rinse, followed by 5 mL of distilled water to remove chemical remnants from the canal system.

Root canals were biomechanically prepared using ProTaper rotary system (Dentsply Sirona Endodontics, Ballaigues, Switzerland) according to the manufacturer’s instructions up to a size of F3. Then the taper of the canal was removed using Peeso reamer size 1–3 (Mani Inc, Tokyo, Japan). Irrigation was performed using a 27-gauge side vented needle (Vista Dental Inc, Racine, WI, USA), which was inserted 1 mm short of the working length. Following the final irrigation regimen, the root canals were dried using paper points (Dentsply Sirona). Samples in each group were then subdivided into two groups (*n* = 12, each) based on sealers used, AH Plus (DeTrey) versus BioRoot RCS (Septodont).

The teeth were horizontally sectioned at the middle third using hard tissue microtome (Leica Biosystems, Nußloch, Germany) under continuous water cooling to obtain a slice of 2.0 ± 0.1 mm thickness. Sealers were then mixed according to manufacturer’s instructions and were filled into the root canals. Specimens were placed in 100% humidity for 48 h to ensure complete setting of the sealers.

Every sectioned tooth sample which was filled with sealer was subjected to POBS measurement. The root canal diameter, as well as the thickness of each slice, was recorded using a digital calliper. Push out test was carried out using a universal testing machine (Instron, Norwood, MA, USA). The force was applied in an apico-coronal direction at a crosshead speed of 1 mm/min using stainless steel plungers of 0.9 mm diameter positioned so that it will contact only the filling material. The maximum force (F) applied at bond failure was recorded in Newton.

The POBS was calculated in MPa using the formula:

POBS (MPa) = Force (N)/adhesion surface area (mm^2^)

The adhesion surface area was calculated by the following equation:

Adhesion surface area (mm^2^) = 2 × π × r × h

where r is the root canal radius, π is the constant 3.14 and h is the thickness of the root slice.

### 2.5. Statistical Analysis

Data was analysed using one-way analysis of variance (ANOVA) followed by Tukey’s honest significant difference (HSD) test. The analysis was undertaken using SPSS software, version 20 (SPSS Inc., Chcago, IL, USA). A *p* value < 0.05 was considered to be statistically significant (95% confidence level).

## 3. Results

### 3.1. FTIR Analysis

Compositional analysis revealed that amide III: phosphate ratio decreased in all groups compared to immersion in the inert physiological saline solution, except for Dual Rinse HEDP in saline, in which the ratio increased (Table 1). The maximum decrease was seen in the groups with pure NaOCl without the addition of HEDP. The HEDP counter-acted this effect in a dose-dependent manner. In the 2.5% NaOCl solution, the NaOCl effect was abolished, while in the 5.25% NaOCl solution there was merely a reduction in the amide III: phosphate (Table 1).

In carbonate: phosphate ratio, only the pure 5.25% NaOCl showed an effect that differed from the inert control solution (Table 1).

**Figure 1:**
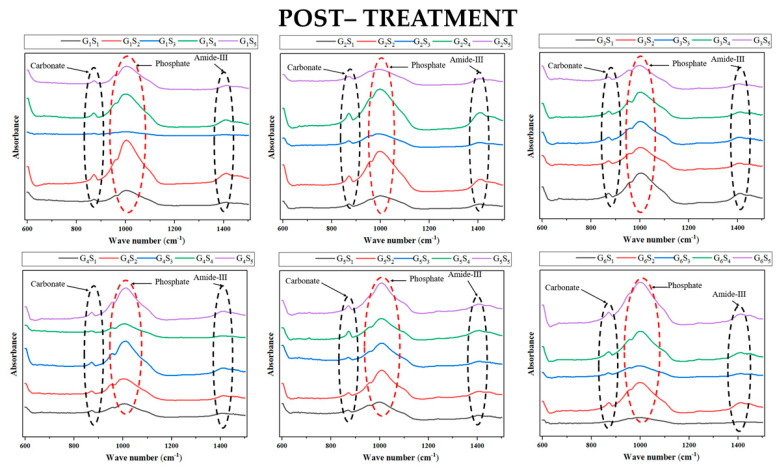
Raw data; FTIR spectra of all the samples (5 samples per group/irrigant: S_1_ to S_5_) after 30 min of exposure to test and control irrigants. G_1_ = 2.5% NaOCl, G_2_ = 5.25% NaOCl, G_3_ = 9% Dual Rinse HEDP in 2.5% NaOCl, G_4_ = 9% Dual Rinse HEDP in 5.25% NaOCl, G_5_ = 9% Dual Rinse HEDP in physiological saline solution, G_6_ = physiological saline solution (control). Note the more homogeneous appearance of the phosphate peaks in the groups irrigated with HEDP (G_3–5_).

The raw data (Figure 1) revealed a considerable variance in overall peak heights between samples, with the specimens treated with HEDP (G3–G5) showing the more consistent spectra.

### 3.2. Push-Out Bond Strength Analysis

**Figure 2:**
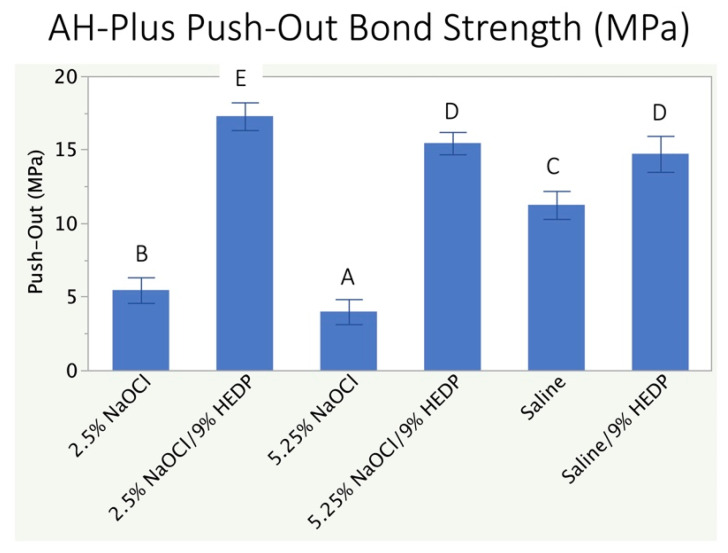
Dislodgment resistance of the epoxy resin sealer under investigation (AH Plus) when root canals were irrigated during and after instrumentation with test and control solutions, with or without the addition of etidronate (Dual Rinse HEDP). Identical superscript letters indicate that there was no difference between data sets at the 5% level (one-way ANOVA, Tukey’s HSD).

When comparing the dislodgment resistance of the AH Plus sealer between groups, all the irrigation solutions caused significant differences between each other (*p* < 0.05) except 5.25% + Dual Rinse HEDP group and Saline + Dual Rinse HEDP (Figure 2). The statistically highest POBS was noted in the group irrigated with 2.5% NaOCl + Dual Rinse HEDP (17.3 ± 1.0 MPa), and the least bond strength was seen with pure 5.25% NaOCl (4.0 ± 0.8 MPa).

**Figure 3:**
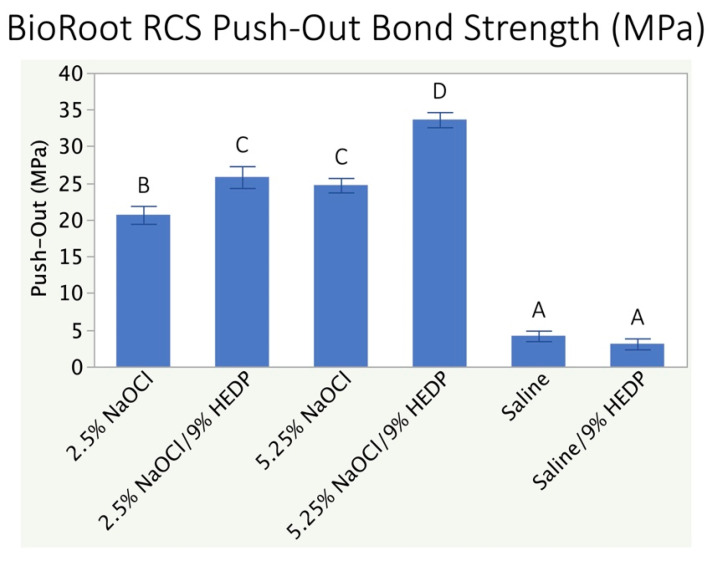
Dislodgment resistance of the hydraulic CaSi sealer under investigation (BioRoot RCS). Identical superscript letters indicate that there was no difference between data sets at the 5% level (one-way ANOVA, Tukey’s HSD).

When comparing the POBS values for BioRoot RCS, no significant differences were noted between 2.5% NaOCl + Dual Rinse HEDP and 5.25% NaOCl, as well as between Saline and Saline + HEDP (Figure 3). However, 2.5% NaOCl alone and 5.25% NaOCl + Dual Rinse HEDP showed a significant statistical difference (*p* < 0.05) between each other and also to the other groups. The maximum value of POBS was noted in 5.25% NaOCl + Dual Rinse HEDP (33.6 ± 1.0 MPa), and the lowest bond strength was noted with saline + HEDP (3.1 ± 0.7 MPa).

## 4. Discussion

Rejecting all null hypotheses, this study showed that the concentration of the NaOCl solution has a dose-dependent effect on the organic content of the root canal wall as assessed by FTIR, and that this effect also reveals itself in the adhesiveness of the two sealer types under investigation. Irrigant effects on sealer adhesiveness were not minor, as they resulted in a fold-factor increase or decrease in sealer adhesion compared to the control treatment utilizing a chemically inert physiological saline solution (Figure 2 and Figure 3).

This study is limited by its in vitro nature. However, since extracted human teeth were used, the results may still have clinical value. The dislodgment resistance of sealers in human root canals has been shown to be directly connected to their prevention of fluid leakage through the same [36]. Sealability, while considered unimportant for a while, has regained attention, because a root filling system that lost its ability to seal root canals of extracted human teeth over time [37] also showed catastrophic clinical long-term results and has been withdrawn from the market [38].

A further limitation of this work is that the dentin samples were treated with the experimental solutions for 30 min for FTIR analysis. This is because our pilot work demonstrated that this time of exposition was necessary to show clear-cut differences in amide III: phosphate ratios, which is in line with published information [33]. However, this also underlines that FTIR and dislodgment resistance testing are not perfectly compatible, because the former requires more specimen preparation and prolonged exposure times, while the latter can be performed in a simulated clinical set-up in root canals of extracted teeth. Nevertheless, FTIR does give some interesting information regarding the exposition of the collagenous dentin matrix effected by decalcifying agents versus the deproteinization caused by NaOCl. Under current conditions the pure NaOCl solution caused a reduction of the amide III/phosphate ratio. The addition of HEDP to the NaOCl counter-acted that. The interpretation of this is that HEDP removed some CaP from the dentin surface, thus keeping the pre-operative or native equilibrium between the organic and inorganic dentin components (Table 1). This would also explain why HEDP was beneficial for the adhesion of both sealer types, despite their apparently different interaction with the root canal wall. It is also to be noted that the inorganic content of the dentin as indicated by the carbonate: phosphate ratio remained unaffected by the irrigants under investigation, except for the 5.25% NaOCl, which caused a significant increase in this ratio (Table 1). The statistically significant increase in the ratio before and after treatment with 5.25% NaOCl cannot be justified. It reveals that FTIR may not be the suitable method to assess the change in inorganic content in tooth samples. Further research needs to be executed in this area to gain more insight and clarity regarding this.

The FTIR results of the present study is in accordance with Tartari et al. [33,39] who demonstrated that, with an increase in NaOCI concentration and contact time, there was significantly increased dissolution of organic matter and dentin collagen with reduction in the amide III: phosphate ratio [39], and that the use of HEDP reduced the carbonate: phosphate ratio as seen in FTIR analysis [33]. Tartari and co-workers also investigated the effects of several decalcifying agents (9% and 18% HEDP, 5% and 10% tetrasodium EDTA, 17% trisodium EDTA, and 0.5% and 2.0% peracetic acid) alone and in alternation with NaOCl on the organic and inorganic components of dentin using FTIR. Srivastava et al. [40] evaluated the effect of 0.9% saline, 5.25% NaOCl + 17% EDTA, 17% EDTA + 2% chlorhexidine and 17% EDTA + 3% green tea extract on the POBS AH-Plus and BioRoot RCS sealers. Their results suggested that BioRoot RCS exhibited significantly higher POBS compared to AH Plus, which is in accordance with the results of the current study. Alfawaz et al. [41] evaluated the bond strength of BioRoot RCS as compared to EndoSequence BC, MTA Fillapex, AH Plus, and Pulp Canal Sealer after treating the specimens with 5.25% NaOCl, 17% EDTA, and SmearClear. Results suggested that BioRoot RCS had significantly higher POBS to root dentin than the other sealers. This result was in accordance with the current study in which BioRoot RCS showed higher bond strength values. Donnermeyer et al. [42] compared the dislodgement resistance of Total Fill BC, Endo CPM, BioRoot RCS with AH Plus sealers. The root canals were treated with 3% NaOCl followed by 17% EDTA. Under their study conditions POBS of AH Plus sealer was found to be significantly higher than all the calcium silicate-containing sealers. This result is in contrary to the results obtained in the current study, in which higher POBS values were observed with BioRoot RCS. The difference in these results could be attributed to the variations in the volume of NaOCl used, preparation taper and the use of core material. In the current study, samples filled with AH Plus sealer had the least POBS when treated with 5.25% NaOCl, which could be attributed to property of NaOCl acting as an organic solvent, which dissolves the collagen fibers on the surface and from the sub-surface of the dentin [43]. This result is in accordance with Vilanova et al. [44]. The compositional analysis performed for the same also revealed maximum decrease in the amide III: phosphate ratio for both NaOCl groups when used alone. This finding also correlates and justifies the poor bond strength values of AH Plus sealer to root canal dentin after the irrigation regimen consisting of NaOCl alone. The highest POBS value was seen with 2.5% NaOCl + Dual Rinse HEDP group. This could be attributed to the amino group exposure in dentinal collagen and simultaneous reduction in smear layer content and debris by mild chelating action rendered by HEDP, which allows increased covalent bond formation between epoxy group and amino groups, resulting in an improved bond of AH Plus to radicular dentin [25].

In the root canals filled with BioRoot RCS, POBS did not seem to be affected by the reduction in organic content at the dentin surface by 5.25% NaOCl. The statistically highest POBS, however, was seen with 5.25% NaOCl + Dual Rinse HEDP. This could be attributed to the continuous chelating action, which enabled the dissolution of organic components of dentin, as well conditioning of the inorganic part of dentin. This leads to the reduction of smear layer and smear plugs improving micromechanical retention and thereby yielding a higher bond strength to root canal dentin [31]. Again, it would appear that HEDP when added to NaOCl keeps the dentin “clean” and in its native state, with the hydraulic CaSi sealer under investigation less affected by dentin deproteinization than the epoxy resin, with even a slightly but significantly better adhesiveness observed using 5.25% NaOCl versus 2.5% (Figure 3). The combined treatment dentin with NaOCl and HEDP has been shown to result in partially degraded but mineralized collagen fibres [45]. This appears to provide an ideal surface for the hydraulic CaSi sealer under investigation to bind to the canal wall. The least POBS was seen in saline + Dual Rinse HEDP group as there was negligible dissolution of organic as well as inorganic component and therefore limiting bonding between BioRoot RCS and root canal dentin.

In conclusion, the combined use of NaOCl and Dual Rinse HEDP appears to result in a homogenous distribution of organic and inorganic components on the treated surface [45]. The result of this study also suggests that the best irrigation sequence while using an epoxy resin-based sealer (AH Plus) and calcium silicate-based sealer (BioRoot RCS) were obtained after using a combined application of 2.5% and 5.25% NaOCl with Dual Rinse HEDP, respectively. Hence, the observation that not only the chelator, but also the deproteinizing agent used for root canal irrigation has a decisive effect in this context is something to consider when striving to match irrigation and obturation strategies in order to achieve an ideal performance of root canal sealers [46]. It is noteworthy that this concept is not even followed or advocated by the companies producing the respective sealers. As indicated by Zancan and co-workers [46] and piloted by De-Deus and co-workers [12], the conditioning of the canal wall plays a decisive role in the subsequent canal wall adhesion and performance of root canal sealers. While there are whole scientific journals dedicated to Adhesive Dentistry, the focus has been on coronal restorations, i.e., the adhesion of restorative materials to the crown of the tooth. The root canal system has not been considered much in this context. As this communication and previous works strongly suggest, however, it should be.

## Figures and Tables

**Table 1:** FTIR ratios (values are multiplied by 1000) before (pre) and after (post) 30 min of immersion in the different irrigants.

Irrigant	Amide III/Phosphate		Carbonate/Phosphate	
	Pre	Post	Pre	Post
Saline = control	7.4 ± 0.6 ^A^	7.0 ± 1.3 ^A^	22.0 ± 1.9 ^a^	21.1 ± 2.2 ^a^
9% HEDP in saline	7.9 ± 0.6 ^A^	8.0 ± 1.1 ^A^	23.1± 4.0 ^a^	21.7± 2.2 ^a^
2.5% NaOCl	7.3 ± 0.7 ^A^	0.4 ± 0.2 ^B^	23.6 ± 1.0 ^a^	22.2 ± 1.3 ^a^
9% HEDP in 2.5% NaOCl	7.6 ± 1.1 ^A^	6.1 ± 2.2 ^A^	25.2 ± 0.7 ^a^	21.1 ± 1.6 ^a^
5.25% NaOCl	7.3 ± 0.8 ^A^	0.7 ± 0.5 ^B^	21.9 ± 1.7 ^a^	29.6 ± 3.0 ^b^
9% HEDP in 5.25% NaOCl	7.5 ± 0.7 ^A^	2.2 ± 1.2 ^B^	22.0 ± 2.1 ^a^	18.2 ± 3.4 ^a^

Means ± standard deviations. Identical superscript letters indicate that there was no statistically significant difference between data sets within that column (one-way ANOVA, Tukey’s HSD), i.e., that there was no difference from the control treatment with an inert saline solution.

## Data Availability

Data are available upon request by the authors.
